# Sensitivity of Malignant Peripheral Nerve Sheath Tumor Cells to TRAIL Is Augmented by Loss of NF1 through Modulation of MYC/MAD and Is Potentiated by Curcumin through Induction of ROS

**DOI:** 10.1371/journal.pone.0057152

**Published:** 2013-02-21

**Authors:** David E. Reuss, Jana Mucha, Christian Hagenlocher, Volker Ehemann, Lan Kluwe, Victor Mautner, Andreas von Deimling

**Affiliations:** 1 CCU Neuropathology German Cancer Research Center (DKFZ), Heidelberg, Germany; 2 Department Neuropathology, Institute of Pathology, University of Heidelberg, Heidelberg, Germany; 3 Department of Neurology, University Hospital Hamburg-Eppendorf, Hamburg, Germany; 4 Institute of Pathology, University of Heidelberg, Heidelberg, Germany; University of Sherbrooke, Canada

## Abstract

Malignant peripheral nerve sheath tumor (MPNST) is a rare aggressive form of sarcoma often associated with the tumor syndrome neurofibromatosis type 1 (NF1). We investigated the effects of tumor necrosis factor-related apoptosis inducing ligand (TRAIL) on NF1 associated MPNST and determinants of TRAIL sensitivity. MPNST cell lines with complete neurofibromin deficiency were sensitive to apoptotic cell death induced by TRAIL whereas MPNST cells with retained neurofibromin expression or normal human Schwann cells were resistant. Increased sensitivity to TRAIL was associated with overexpression of death receptors, especially DR5. Re-expression of the GAP related domain of neurofibromin (NF1-GRD) suppressed DR5 expression and decreased sensitivity to TRAIL. We show that death receptor expression and TRAIL sensitivity critically depend on c-MYC and that c-MYC amounts are increased by MEK/ERK and PI3K/AKT signalling pathways which are suppressed by neurofibromin. Furthermore PI3K/AKT signalling strongly suppresses the MYC-antagonist MAD1 which significantly contributes to TRAIL sensitivity. Re-expression of the NF1-GRD decreased c-MYC and increased MAD1 amounts suggesting that neurofibromin influences TRAIL sensitivity at least in part by modulating the MYC/MAX/MAD network. The phytochemical curcumin further increased the sensitivity of neurofibromin deficient MPNST cells to TRAIL. This was presumably mediated by ROS, as it correlated with increased ROS production, was blocked by N-acetylcysteine and mimicked by exogenous ROS.

## Introduction

Malignant peripheral nerve sheath tumors (MPNST) are highly malignant tumors of the Schwann cell lineage, which either arise from peripheral nerve or in extraneural soft tissue. MPNST are rare in the general population. However, patients with neurofibromatosis type I (NF1) have a lifetime risk of 8% to 13% to develop MPNST. About 50% of MPNSTs are associated with NF1 and these tumors are the major cause of reduced life expectancy of NF1 patients [1], [2]. MPNST in NF1 patients harbour a somatic *NF1* gene mutation in addition to the underlying germline mutation [3], [4]. *NF1* gene mutations have been found also in a subset of sporadic MPNST [5], [6]. The *NF1* gene product neurofibromin functions at least in part as GTP-ase activating protein (GAP) for RAS proteins via its GAP related domain (NF1-GRD). Neurofibromin promotes the conversion of active GTP bound RAS to the inactive GDP bound form. Therefore loss of function of neurofibromin favours the active status of RAS proteins [7], [8]. MPNST are highly resistant towards conventional radio- and chemotherapy which act predominantly by inducing apoptosis. Downstream of RAS there are at least two pathways involved in regulation of apoptosis, the RAF/MEK/ERK and the PI3K/AKT pathways. As MPNST lack sensitivity for apoptosis induction by conventional chemotherapeutics, novel substances which trigger apoptosis may be efficient. In this context the TNF-alpha related apoptosis inducing ligand (TRAIL) is of special interest, as it has been shown to induce apoptosis effectively in cancer cells but not in normal cells [9]. However, not all tumor cells are sensitive to TRAIL and resistance of tumor cells is a major obstacle for TRAIL based therapy. In cellular transformation models oncogenic RAS has been shown to induce TRAIL susceptibility at least in part by upregulation of death receptors DR4 and DR5 [10], [11]. Due to the lack of efficient therapeutics for MPNST and the potential link between loss of function of neurofibromin, RAS signalling and TRAIL sensitivity, we were interested in evaluating the effects of TRAIL on MPNST cells.

## Materials and Methods

### Cell culture

1507.2 cells were newly established from a NF1 associated MPNST. S462 cells have been described before [12], ST88-14, NFS-1, STS-26T were provided from Dr. Holtkamp (Charité Berlin, Germany). All cell lines were cultured in DMEM Glutamax-I 4500 g/l glucose (Invitrogen, Karlsruhe, Germany) with 10% FBS and 1% penicillin/streptomycin (Invitrogen, Karlsruhe, Germany) and was incubated at 37°C in a humidified atmosphere containing 10% carbon dioxide. Human Schwann cells (HSC) were obtained from ScienCell and cultured in medium containing DMEM 10% FBS, 10 ng/ml Heregulin and 2 µM Forskolin and 1% penicillin/streptomycin. HSC used as controls were cultured for 48 h in the same medium as the MPNST cell lines.

### Ethics Statement

This project was approved by the ethics committee of the University Hospital Hamburg-Eppendorf. Investigations were carried out with written consent of the patient.

### Reagents

MEK-inhibitor U0126 was from Promega (Madison, Wis., USA). PI3K-inhibitor Ly294002, curcumin, genistein, capsaicin and resveratrol were from Calbiochem (San Diego, CA, USA). Recombinant human TRAIL was from Peprotech (Rocky Hill, NJ, USA). N-acetylcysteine was from (Sigma, St. Louis, MO, USA).

### Crystal violet viability assay

Cells were seeded in 6- or 12-well plates and grown to 70–80% confluence before treatment with TRAIL. After incubations cells were washed two times with PBS and fixed with cold methanol for 20 min at −20°C. 0.4% crystal violet solution (Sigma) was added and incubated for 30 min at room temperature. After extensive washing with water plates were dryed. Plates were destained with 0.1 M sodium citrate in 50% ethanol for 30 min with agitation. Absorbance was measured at 550 nm in a plate reader. The calculated additive effect was evaluated by the fractional product method: f_u_(1,2) = f_u_(1) X f_u_(2) [13]. The formula allows a prediction of the effect of the cotreatment with two agents on the basis of the assumption that they do not interact or cooperate. If the observed percentage of surviving cells was below the calculated product increased TRAIL sensitivity was considered.

### Antibodies

DR4 and DR5 antibodies were obtained from abcam (abcam, Cambridge, UK); neurofibromin (D) was from Santa Cruz (Santa Cruz, CA, USA); phospho-ERK1 and –ERK2 (T202/Y204 and T185/Y187) from (R&D Systems Inc., Minneapolis, MN, USA); GFP, MAD1, α-tubulin and β-Actin from Sigma; phospho-AKT, phospho-IκBα, XIAP, MCL-1, PARP, Caspase 8, Caspase 9 from Cell Signaling Technology (Beverly, MA, USA).

### SDS-PAGE and Immunoblotting

Cells were lysed in NuPAGE® LDS Sample Buffer containing glycerol (30–60%) and lithium dodecyl sulfate (7–13%) (Invitrogen). β-mercapto-ethanol (Sigma) was added and samples were denatured at 95°C for 5 min and electrophoretically separated on 4%–12% BIS-TRIS gels (Invitrogen). Proteins were blotted onto nitrocellulose membranes (Invitrogen). After blocking (5% milk powder, 0.05% Tween20 in PBS or 2% BSA, 0.1% Tween20 in PBS) at room temperature for 1 h the membranes were incubated overnight at 4°C with primary antibody in blocking solution. Staining with secondary horseradish peroxidase–conjugated anti-rabbit or anti-mouse antibodies (Amersham Biosciences, Braunschweig, Germany) for 1 h at room temperature was followed by immunodetection with Western Blotting Detection System ECL (KPL, Inc., Gaithersburg, Maryland, USA). Densitometry analysis of bands was performed with Image J software (National Institutes of Health (NIH), Bethesda, MD, USA).

### Transfections

Transfections of 1507.2 cells were performed with LONZAs (Basel, Swiss) nucleofector using “basic primary neuron solution”; program T30. 2×10^6^ cells were nucleofected with 2 µg of Plasmid-DNA or 1.5 µg siRNA (Dharmacon, Inc., Lafayette, CO, USA) and seeded in 12-Well plates. siRNA transfected cells were treated with 100 ng/ml TRAIL after 48 h. Plasmid DNA transfected cells were cultured for further 48 h before treatment with 100 ng/ml TRAIL. The pEGFP-NF1-GRD plasmid was kindly provided by Dr. Klaus Scheffzek. The empty control vector was from Clontech (Mountair View, CA, USA).

### Protein array

Proteome Profiler Human Apoptosis Array Kit from R&D Systems was used according to manufactures instructions.

### Flow cytometry

Apoptotic cells were assessed by flow cytometry with PI-method. For detection of apoptotic cells a FACS Calibur flow cytometer equipped with a 488 nm air cooled argon laser (Becton & Dickinson, Cytometry Systems, San Jose, CA, USA) was used with filter combinations for propidium iodide. For analyses and calculations the Cellquest program (Becton & Dickinson, Cytometry Systems, San Jose, CA, USA) was used. Each histogram and dot plot represented 10,000 cells. After cell preparation according to Nicoletti with modifications [14], [15] measurements were acquired in Fl-2 in logarithmic mode and calculated by setting gates (M-1) over the first three decades to detect apoptotic cells. Percentage of specific apoptosis was calculated as follows: 100 X [TRAIL apoptosis (%) - spontaneous apoptosis (%)/100% - spontaneous apoptosis (%)] [16].

### Measurement of ROS

Intracellular ROS generation was assessed with dichlorodihydrofluorescein diacetate (DCF-DA) that was purchased from Invitrogen. To detect intracellular reactive oxygen species (ROS), cells were preincubated with 10 µmol/L DCF-DA for 30 min at 37°C in the dark. After washing with PBS cells were treated with curcumin or H_2_O_2_. After 60 min of incubation, the increase in fluorescence resulting from oxidation of DCF-DA to DCF was measured using a fluorometer (FLUOstar Omega, BMG Labtech, Offenburg, Germany). The mean fluorescence intensity at 530 nm was calculated using triplicate measurements.

### Statistical analysis

The probability of significant difference between viability of cells was done using Student's t test for paired data (two-tail). Differences were considered significant if P<0.05 and highly significant if P<0.01.

## Results and Discussion

### NF1 associated MPNST cell lines are sensitive to TRAIL

**Figure 1:**
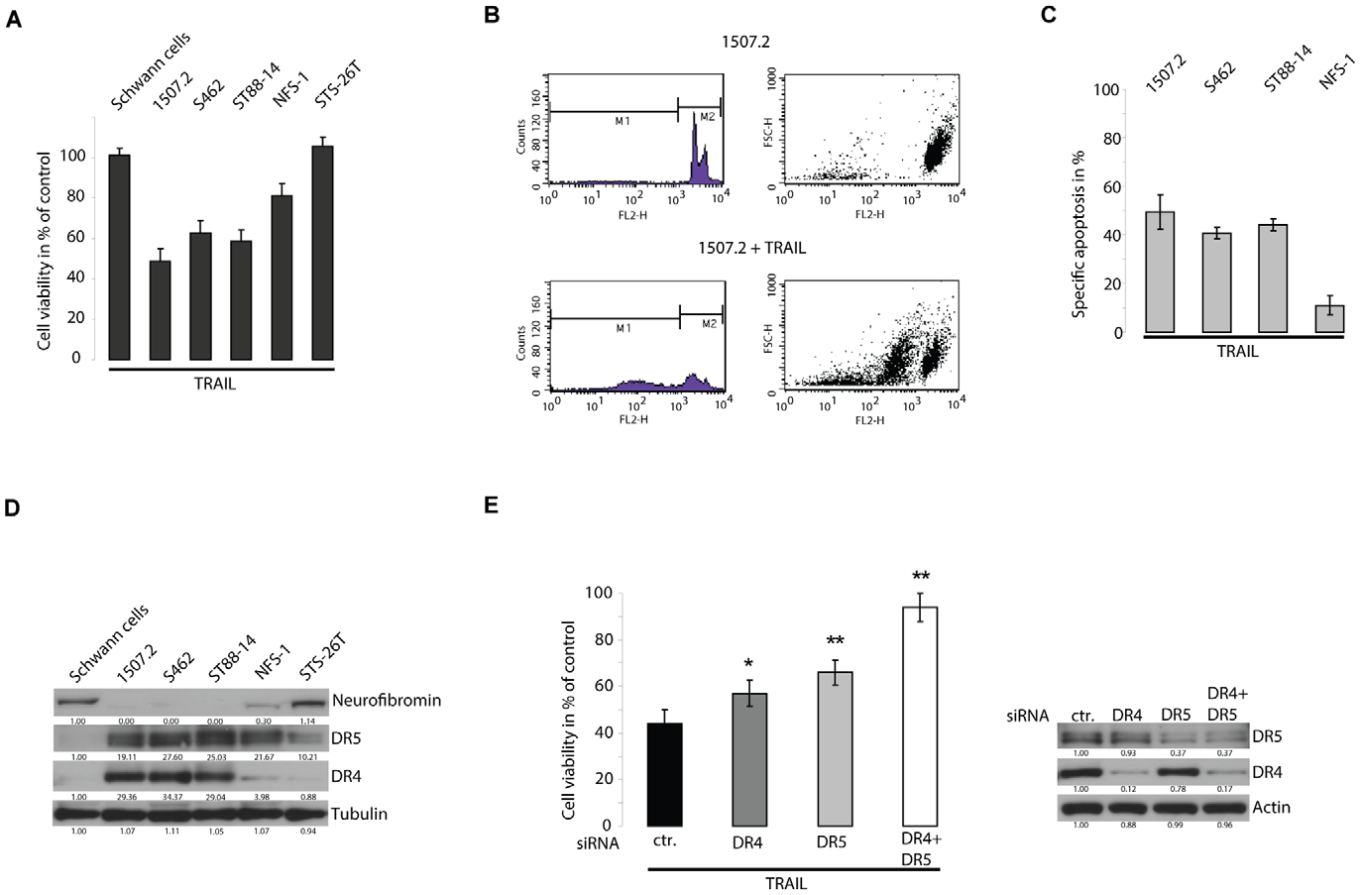
NF1 associated MPNST cell lines are sensitive to TRAIL and overexpress death receptors. A, Normal human Schwann cells, NF1 associated cell lines 1507.2, S462, ST88-14, NFS-1 and sporadic cell line STS-26T were treated with 100 ng/ml TRAIL for 20 h. Viability was analyzed by crystal violet assay and is expressed as percent of untreated control. B, FACS analysis of PI-stained cells showing TRAIL-induced apoptosis. C, Specific apoptosis induced by TRAIL in MPNST cell lines. Immunoblots show upregulation of death receptors in neurofibromin deficient cell lines. E, 1507.2 cells transfected with control siRNA or siRNA targeting DR4 and DR5 were treated with 100 ng/ml TRAIL for 20 h. Corresponding immunoblots show death receptor expression (DR4, DR5). D, E, The values below the bands are the relative densities. A, C, E, Results are expressed as mean ± s.d. of three independent experiments (n = 3).

The effects of TRAIL were investigated on 5 different MPNST cell lines (1507.2, S462, ST88-14, NFS-1 and STS-26T) and on normal human Schwann cells. 1507.2, S462, ST88-14 and NFS-1 cell lines derive from MPNST arising in NF1 patients, the STS-26T cell line derives from a sporadic MPNST. Normal human Schwann cells are resistant towards a treatment with 100 ng/ml TRAIL for 20 h. The 5 different MPNST cell lines displayed a variable sensitivity to TRAIL. The 4 NF1 associated MPNST cell lines were sensitive to TRAIL. 1507.2 was the most sensitive cell line with a mean viability loss of 52%, S462 and ST88-14 showed a mean decrease in viability of 37% and 41% respectively. NFS-1 cells had a mean loss of viability of 20%. Sporadic MPNST derived STS-26T cells were completely resistant to TRAIL (Figure 1A). STS-26T cells remained resistant even if a concentration of 1000 ng/ml TRAIL was used or the treatment was prolonged to 48 h (data not shown). Flow cytometry analysis using propidium iodide staining confirmed that reduction of cell viability upon TRAIL treatment was due to induction of apoptosis (Figure 1B, C).

### NF1 associated MPNST cell lines overexpress death receptors

Neurofibromin is a potent inhibitor of RAS signalling which has been implicated in both sensitivity as well as resistance to TRAIL [10], [11], [17]. Therefore the potential impact of neurofibromin on the sensitivity to TRAIL in MPNST cells was analysed. Immunoblot analyses using a C-terminal antibody showed complete deficiency for full length neurofibromin in NF1 associated 1507.2, S462 and ST88-14 cells. NFS-1 cells showed markedly reduced levels. The sporadic STS-26T cells as well as normal human Schwann cells express neurofibromin (Figure 1D). In these cell lines neurofibromin expression was inversely correlated to TRAIL sensitivity. Cell survival upon treatment with TRAIL depends on different regulators. The abundance of death receptor expression is one of the determinants for TRAIL sensitivity [18], [19].

Oncogenic RAS has been shown to influence death receptor expression [10], [11]. Therefore the expression of death receptors was analysed in MPNST cells. Compared to normal human Schwann cells DR5 was strongly overexpressed in MPNST cells in which neurofibromin was lost, whereas the cell line with retained neurofibromin exhibited lower expression of DR5. Expression of DR4 was also upregulated in 1507.2, S462 and ST88-14 cells (Figure 1D). We hypothesised that sensitivity to TRAIL is increased through death receptor upregulation driven by loss of neurofibromin.

### Overexpression of death receptors is essential for TRAIL sensitivity

To determine the impact of death receptor expression on TRAIL sensitivity in a neurofibromin deficient cell line, we transfected 1507.2 cells with pools of specific siRNA against DR4, DR5 or both. 48 h after transfection cells were treated with TRAIL. Compared to control siRNA transfected cells, knockdown of each death receptor lead to a significantly reduced sensitivity to TRAIL. When both receptors were knocked down simultaneously cells became almost resistant to TRAIL (Figure 1E). This result suggests that the high expression of death receptors in 1507.2 cells is essential for their susceptibility to TRAIL.

### TRAIL sensitivity and death receptor expression are regulated by serum

**Figure 2:**
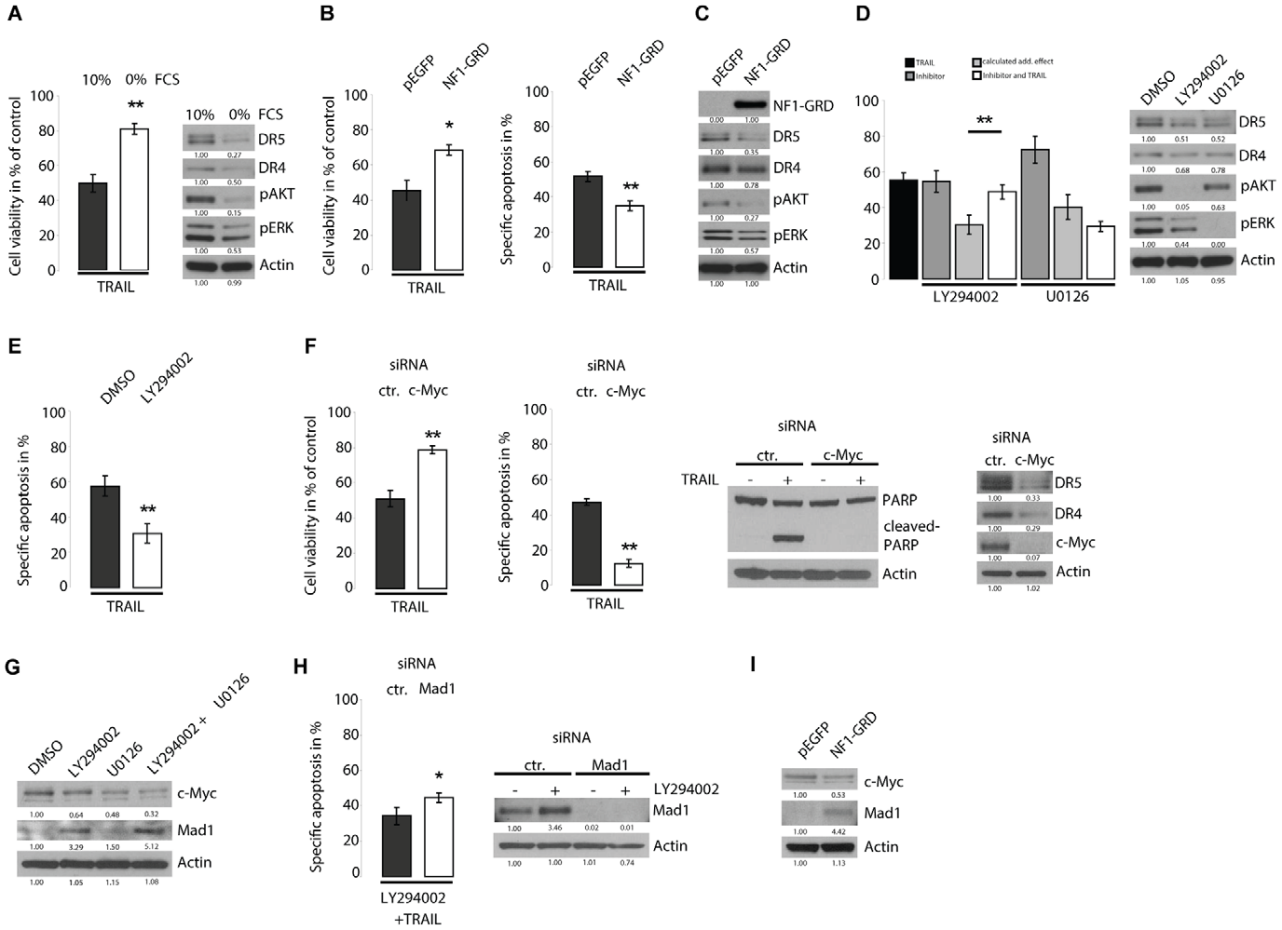
Loss of NF1 contributes to TRAIL sensitivity by PI3K and ERK mediated modulation of c-MYC and MAD1. A, 1507.2 cells were cultured with or without serum for 24 h and subsequently treated with 100 ng/ml TRAIL for 20 h. Corresponding immunoblots show reduced phosphorylation of ERK and AKT as well as downregulation of death receptors under serum free conditions. B, Re-expression of the NF1-GRD in 1507.2 cells reduced the sensitivity to TRAIL concomitant with downregulation of DR5, pAKT and pERK (C). D, 1507.2 cells were pretreated with DMSO or the PI3K inhibitor LY294002 (20 µM) or the MEK inhibitor U0126 (20 µM) for 24 h and subsequently treated with 100 ng/ml TRAIL for 20 h. The calculated additive effect of cotreatment (TRAIL and inhibitor) on cell viability was determined and compared with the actually observed effect. Corresponding immunoblots show phosphorylation levels of ERK and AKT and expression of death receptors (DR4, DR5). E, Specific apoptosis of 1507.2 cells treated with LY294002. F, siRNA mediated knockdown of c-MYC reduced sensitivity to TRAIL and downregulated death receptors. G, Immunoblots of 1507.2 cells treated with DMSO, LY294002, U0126 or LY294002 together with U0126 showing levels of c-MYC and MAD1. H, siRNA mediated knockdown of MAD1 partly rescued TRAIL sensitivity of 1507.2 cells treated with LY294002. I, Re-expression of the NF1-GRD in 1507.2 cells reduced the amounts of c-MYC and increased the amounts of MAD1. Asterisks outline values that are different from respective control (* = P<0.05; ** = P<0.01, Student's t-test). Results are expressed as mean ± s.d. of three independent experiments (n = 3).The values below the bands are the relative densities.

In cells with neurofibromin deficiency the termination of RAS signalling is impaired. However, RAS-GTP levels remain to be dependent on activators [20], [21]. To determine the influence of RAS stimulating growth factors on death receptor expression and sensitivity to TRAIL, 1507.2 cells were starved from serum for 24 h and assayed for TRAIL induced cell death. Compared to cells cultured with serum those cultured in serum free medium showed a highly reduced sensitivity to TRAIL (Figure 2A). Immunoblot analysis of the phosphorylation status of ERK and AKT suggested reduced activity of both of these pathways in cells under serum starvation (Figure 2A). In correlation to the reduced sensitivity to TRAIL, 1507.2 cells showed a strong downregulation of DR5 and a less pronounced downregulation of DR4 under serum free conditions (Figure 2A).

### TRAIL sensitivity and death receptor expression are regulated by neurofibromin

To evaluate a possible role of neurofibromin deficiency for death receptor expression and TRAIL sensitivity, 1507.2 cells were transfected with an EGFP-tagged version of the RAS regulating GAP related domain of neurofibromin (NF1-GRD). Using nucleofection technology a transfection rate of 85% was achieved. 1507.2 cells transfected with NF1-GRD were significantly less sensitive to TRAIL compared to EGFP control vector transfected cells (Figure 2B). Flow cytometry analysis confirmed a significant reduction of specific apoptosis induced by TRAIL in NF1-GRD transfected cells (Figure 2B). Immunoblot analyses confirmed re-expression of the NF1-GRD and showed reduced phosphorylation of both ERK and AKT. Consistent with the previous results NF1-GRD expressing cells showed strong downregulation of DR5 whereas DR4 expression was only slightly altered (Figure 2C). This suggests that loss of neurofibromin upregulates DR5 expression and increases TRAIL sensitivity in 1507.2 MPNST cells.

### ERK and AKT signalling contributes to TRAIL sensitivity and death receptor expression

To determine if MEK/ERK or PI3K/AKT pathways are involved in the regulation of death receptor expression and TRAIL sensitivity we treated 1507.2 cells with specific inhibitors of MEK (U0126) and PI3K (LY294002).

Treatment of cells with these inhibitors alone significantly reduced the viability which could be attributed mainly to an inhibition of proliferation (data not shown). To assess significant changes in TRAIL sensitivity the theoretically expected additive effect of co-treatment was calculated by the fraction product method [13] and compared with the actually observed effect. 1507.2 cells pre-incubated with U0126 for 24 h did not show a significant change in sensitivity to TRAIL, however cells treated with LY294002 were significantly less sensitive to TRAIL (Figure 2D). Densitometric analysis of immunoblots showed that DR5 expression was reduced by both inhibitors in association with dephosphorylation of ERK or AKT respectively. DR4 expression was also slightly reduced by the inhibitors (Figure 2D). Flow cytometry analysis confirmed a significant reduction of specific apoptosis induced by TRAIL in LY294002 treated cells (Figure 2E). We tested the PI3K and MEK inhibitors with all NF1 MPNST cell lines (Figure S1A). Although cell line specific differences are evident these experiments show that at least one of the two main downstream pathways of RAS contributes to DR5 expression in all neurofibromin deficient MPNST cell lines tested. Furthermore the sensitivity to TRAIL of 1507.2 and S462 cell lines was significantly reduced by inhibition of PI3K or MEK respectively. The fact that alterations in death receptor abundance in these experiments did not always translate into altered TRAIL sensitivity may be explainable by the multitude of AKT and ERK targets which may contribute to TRAIL sensitivity or resistance (e.g. Inhibitor of apoptosis proteins, Bcl-2 family of proteins).

Importantly, in none of the NF1 associated cell lines inhibition of PI3K lead to an increase in TRAIL sensitivity which sharply contrasts a multitude of studies from other tumor entities. For example cells of glioblastomas, the most common malignant tumor of the central nervous system, are broadly sensitised to TRAIL mediated apoptosis by LY294002 or other inhibitors of the PI3K/AKT pathway [22], [23]. Another example are cells of neuroblastoma in which TRAIL sensitivity is restored through inhibition of PI3K/AKT [24]. In general PI3K/AKT signalling is an important anti-apoptotic survival pathway which regulates multiple pro- and anti-apoptotic proteins. However, reduced sensitivity to TRAIL and downregulation of death receptors after inhibition of PI3K/AKT has been reported before in a HER2-overexpresssing breast cancer cell line [25]. The failure of PI3K/AKT inhibition to increase TRAIL sensitivity in MPNST cells may be explained by the contribution of PI3K/AKT to DR5 expression in all MPNST cell lines analysed.

In an attempt to explain the influence of PI3K and MEK on death receptor expression and TRAIL sensitivity we hypothesised that for several reasons the transcription factor c-MYC may be involved. First, c-MYC has been reported to be an important determinant of TRAIL sensitivity in other tumors [26]. Second, there is evidence for c-MYC regulating both death receptors 4 and 5 [27], [28]. Third, there is increasing evidence for c-MYC activity being modulated by RAS signalling pathways PI3K and MEK through multiple mechanisms including c-MYC protein stabilisation and regulation of the c-MYC antagonist MAD1 [29]–[31].

### c-MYC is a critical determinant of TRAIL sensitivity and death receptor expression

In order to determine if c-MYC is important for TRAIL sensitivity c-MYC was knocked down in 1507.2 cells using specific siRNA. Compared to control siRNA transfected cells, knock-down of c-MYC strongly attenuated the effect of TRAIL on cell viability (Figure 2F). Flow cytometry showed a highly significant reduction of specific apoptosis induced by TRAIL in cells with c-MYC knock-down (Figure 2F). Immunoblot analyses of apoptosis specific PARP cleavage also indexed strong inhibition of apoptosis induction in c-MYC knock-down cells (Figure 2F). Immunoblots confirmed knock-down of c-MYC and showed a strong downregulation of both death receptors (Figure 2F). These results suggest that modulating c-MYC is a possible mechanism to regulate TRAIL sensitivity and death receptor expression in MPNST cells.

### MEK and PI3K signalling modulate c-MYC and MAD1

Different mechanisms have been uncovered of how MEK/ERK and PI3K/AKT signalling may regulate c-MYC including protein stabilisation [32]. Immunoblot analyses of 1507.2 cells treated with PI3K (LY294002) and MEK(U0126) inhibitors alone and in combination revealed a positive additive effect of both pathways on c-MYC protein amounts (Figure 2G). Notably, inhibition of PI3K was less efficient than inhibition of MEK but only inhibition of PI3K reduced TRAIL sensitivity. This result suggested that PI3K may influence c-MYC by an additional mechanism. Indeed immunoblots revealed a strong upregulation of the cellular MYC-antagonist MAD1 in LY294002 treated cells, whereas U0126 alone barely influenced MAD1 levels. Notably, the combined inhibition of PI3K and MEK resulted in the strongest increase in MAD1 amounts (Figure 2G).

### MAD1 suppression by PI3K contributes to TRAIL sensitivity

These results suggested that PI3K supports TRAIL sensitivity by reducing MAD1 levels. MAD1 competes with c-MYC for binding the obligatory transcriptional co-activator MAX thereby regulating c-MYC activity. Furthermore MAD1 acts as transcriptional repressor on target genes for which c-MYC acts as activator [33], [34]. To evaluate a possible contribution of MAD1 suppression in PI3K mediated TRAIL sensitivity we tested whether siRNA mediated knock-down of MAD1 is able to rescue TRAIL sensitivity in 1507.2 cells treated with LY294002. In control siRNA transfected cells LY294002 lead to an upregulation of MAD1 which is abolished in MAD1 siRNA transfected cells (Figure 2H). After treatment with TRAIL cells with MAD1 knock-down showed a significantly higher rate of apoptosis than control cells confirming a role for MAD1 in TRAIL sensitivity (Figure 2H).

Taken together the data suggests that loss of NF1 contributes to TRAIL sensitivity of MPNST cells by modulating the MYC/MAX/MAD network. Consistent with this idea re-expression of the NF1-GRD in 1507.2 cells halves the c-MYC amounts and strongly upregulates those of MAD1 (Figure 2I).

### Curcumin increases the sensitivity of NF1 associated MPNST cells to TRAIL

**Figure 3:**
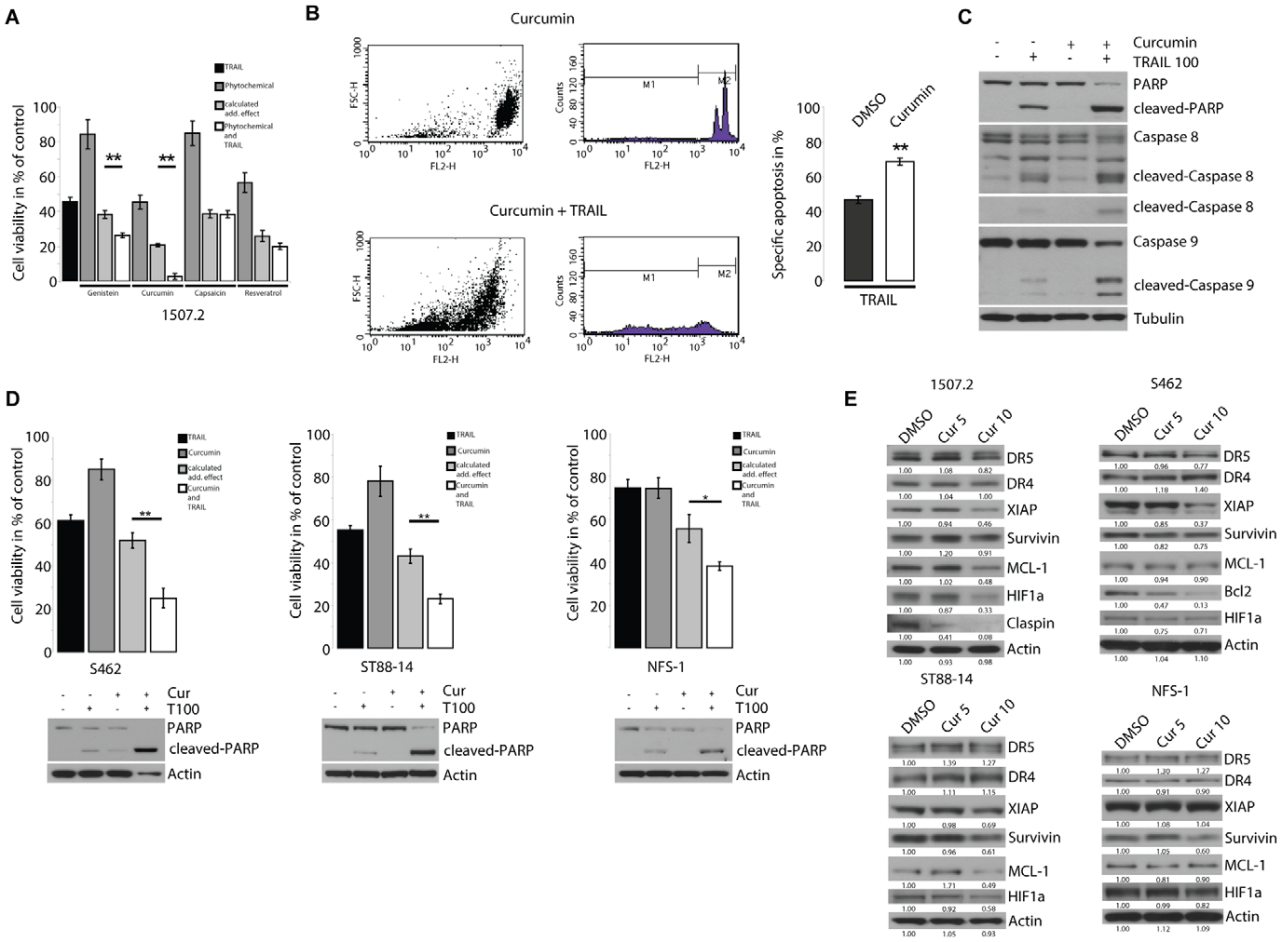
Curcumin increases the sensitivity of NF1 associated MPNST cells to TRAIL. A, 1507.2 cells were pretreated with 10 µM of the indicated phytochemical and subsequently treated with 100 ng/ml TRAIL for 20 h. The calculated additive effect (phytochemical and TRAIL) was determined and compared with the actually observed effect. B, FACS analysis of 1507.2 cells treated with curcumin alone or in combination with TRAIL. C, 1507.2 cells were preincubated with DMSO or curcumin and subsequently treated with 100 ng/ml TRAIL or left untreated. Immunoblots show levels of caspase 8, caspase 9, PARP and their corresponding cleavage fragments. D, S462, ST88-14 and NFS-1 cells were pretreated with DMSO or 10 µM curcumin and subsequently treated with 100 ng/ml TRAIL for 20 h. Viability was analyzed by crystal violet assay. Corresponding immunoblots show PARP and its cleavage fragment. E, Curcumin treatment of NF1 associated MPNST leads to downregulation of anti-apoptotic proteins. 1507.2, S462, ST88-14, NFS-1 cells were treated for 24 h with DMSO, 5 and 10 µM curcumin and analyzed for expression of apoptosis regulating proteins by immunoblotting. BCL-2 expression is only depicted for S462 cells because it was not detectable in 1507.2 and ST88-14 cells. Claspin expression was only detectable in 1507.2 cells. The values below the bands are the relative densities. E, A, B, D, Results are expressed as mean ± s.d. of three independent experiments (n = 3). Asterisks outline values that are different from respective control (* = P<0.05; ** = P<0.01, Student's t-test). The values below the bands are the relative densities.

Even though neurofibromin deficient MPNST cells harbour an intrinsic sensitivity to TRAIL the therapeutic potential of TRAIL alone may be insufficient. However, phytochemicals have been shown to augment TRAIL sensitivity [35]. Therefore phytochemicals were screened for their ability to increase the sensitivity to TRAIL. 1507.2 cells were used for the screen as we expected them to provide the most sensitive readout. 1507.2 cells pre-incubated with subtoxic dose (10 µM) of genistein, curcumin, capsaicin or resveratrol for 24 h were assayed for their relative loss of viability upon treatment with 100 ng/ml TRAIL. Of these agents curcumin increased the sensitivity to TRAIL most significantly (Figure 3A). Flow cytometry analysis showed a highly significant increase in specific apoptosis induced by TRAIL (Figure 3B). Of note, we observed sub G1 peaks of propidium iodide stained cells in the 3rd and 2nd decade already at a 6 h timepoint to almost the same extend as at a 20 h timepoint consistent with apoptotic cell death. Furthermore immunoblot analysis showed increased cleavage of PARP, caspase 8 and caspase 9 in cells treated with curcumin and TRAIL compared to cells treated with TRAIL alone confirming augmented TRAIL mediated apoptosis by curcumin (Figure 3C). Morphological parameters in flow cytometry analysis suggested that cell death occurred in a smaller percentage also outside of the apoptotic cell population explaining the almost complete loss of viability seen in 1507.2 cells upon curcumin and TRAIL treatment. Of note, TRAIL has been shown before to be able to induce necrosis or necroptosis [36].

To evaluate if augmented sensitivity to TRAIL is a cell line specific feature we tested curcumin with all MPNST cell lines and with normal human Schwann cells. Curcumin increased the sensitivity to TRAIL in all NF1 associated MPNST cell lines (Figure 3D) but failed to sensitize STS-26T cells (data not shown). Viability of normal human Schwann cells was not affected by curcumin alone or in combination with TRAIL (data not shown).

### Curcumin downregulates anti-apoptotic proteins

Next we used protein arrays measuring the levels of 35 apoptosis related proteins to get a deeper insight into the effects of curcumin. Protein lysates of 1507.2 cells treated with curcumin for 36 h were compared with those of DMSO treated cells. Array results suggested downregulation of anti-apoptotic XIAP, claspin and HIF1α by curcumin (Figure S1B). Immunoblot analyses were performed to confirm array results. Immunoblotting also revealed downregulation of MCL-1 which was not represented on the array (Figure 3E). Furthermore immunoblotting analyses were performed with S462, ST88-14 and NFS-1 cells treated with curcumin. In S462 cells curcumin downregulated XIAP, survivin and BCL-2, in ST88-14 cells survivin, MCL-1 and HIF1α and in NFS-1 cells survivin and HIF1α (Figure 3E). Notably death receptor expression was only slightly altered by curcumin.

However, ectopic expression of XIAP or MCL-1 failed to rescue 1507.2 cells from cell death induced by the combination of curcumin and TRAIL (data not shown), indicating that downregulation of these proteins is not critical for curcumin to sensitize cells.

### Curcumin influences multiple signaling cascades

**Figure 4:**
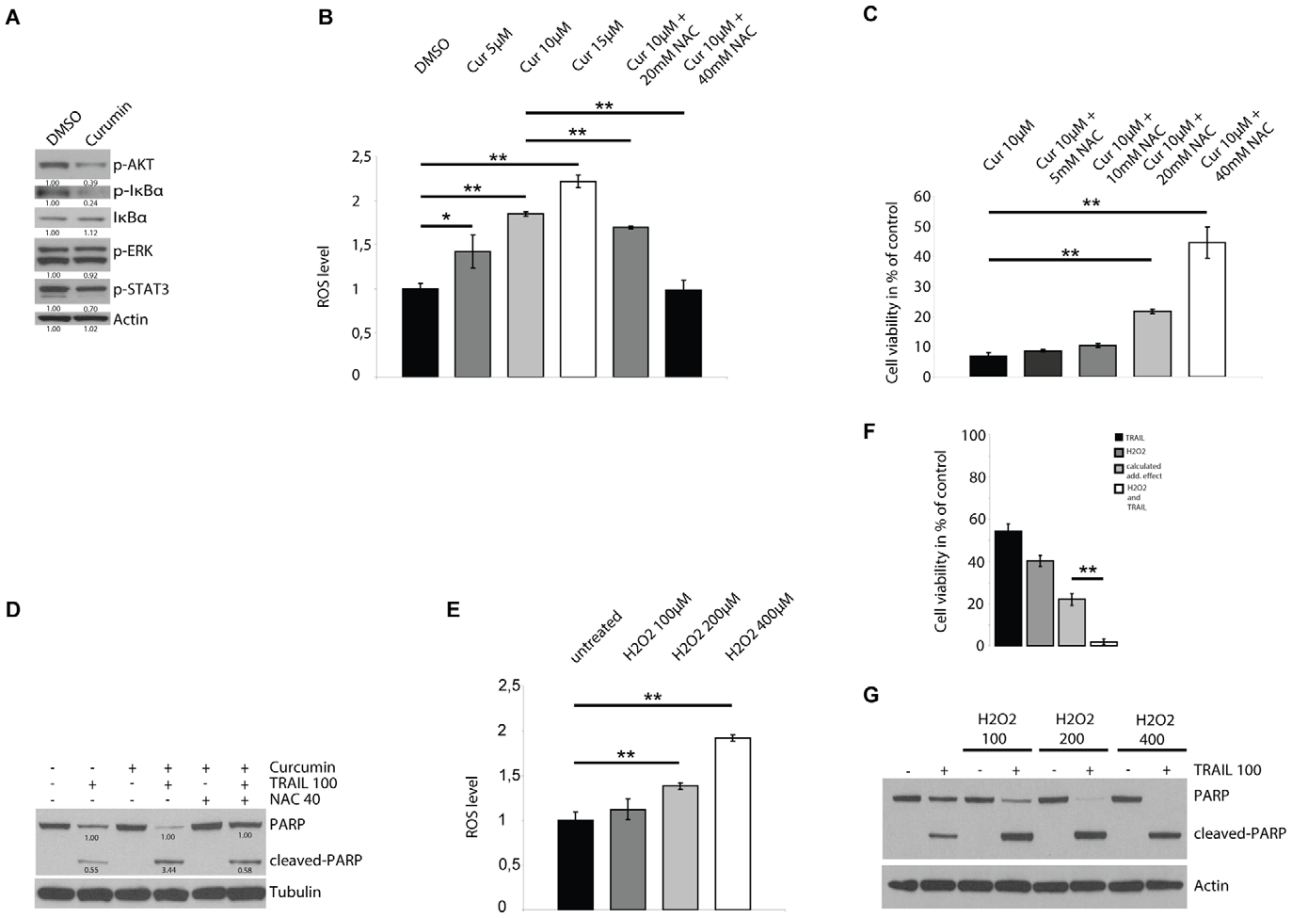
Curcumin potentiates TRAIL sensitivity through induction of ROS. A, Immunoblots of 1507.2 cells treated with curcumin show reduced levels of p-AKT, p-Stat3 and p-IKBa. B, Curcumin induced ROS in 1507.2 cells dose dependently which was blocked by ROS-scavenger N-acetylcystein (NAC). C, H_2_O_2_ increased levels of ROS in 1507.2 cells dose dependently. D, NAC rescued cell viability of TRAIL-treated 1507.2 cells dose dependently. E, NAC abrogated the curcumin-induced enhancement of PARP-cleavage. F, H_2_O_2_ (400 µM) increased the sensitivity of 1507.2 cells to TRAIL. G, H_2_O_2_ increased PARP cleavage of TRAIL treated 1507.2 cells dose dependently. Results are expressed as mean ± s.d. of three independent experiments (n = 3). Asterisks outline values that are different from respective control (* = P<0.05; ** = P<0.01, Student's t-test).

Curcumin is a pleiotropic agent influencing multiple signaling pathways and molecules. To get an overview over the effects of curcumin on signaling we used immunoblots of key phospho-proteins of several signaling pathways previously shown to be influenced by curcumin. Curcumin reduced the phosphorylation of AKT, IκBα and STAT3 while pERK remained unchanged (Figure 4A).

Curcumin has been shown to inhibit NF-κB activation on different levels including IκBα phosphorylation [37], [38]. IκBα acts as an inhibitor of NF-κB by retaining it in the cytoplasm. Various stimuli including growth factors and cytokines rapidly inactivate IκBα by phosphorylation which is mediated by the IKK signaling complex. Activation of NF-κB is reported to correlate with phosphorylation and degradation of IκBα [39]. While immunoblots of 1507.2 cells treated with curcumin showed indeed reduced levels of phosphorylated IκBα, total levels of IκBα however remained almost unchanged (Figure 4A). To clarify whether IκBα plays a role in curcumin potentiated TRAIL sensitivity, we used siRNA to knock-down IκBα and tested if this attenuates the effect of curcumin. 1507.2 cells with knock-down of IκBα did not show a rescue from cell death disfavoring IKK/IκBα as critical target for curcumin (data not shown). Expression of a constitutively active form of STAT3 (STAT3C) also failed to rescue cells from curcumin and TRAIL induced cell death (data not shown).

### Curcumin induces ROS production

Curcumin has also been shown to induce the production of reactive oxygen species (ROS) in tumor cells [40]. ROS is known to play an important role in cell fate decision and TNF alpha mediated cell death and has also been implicated in TRAIL sensitivity [41]. Therefore we investigated whether ROS production is involved in curcumin mediated TRAIL sensitization. Curcumin induced intracellular ROS dose dependently in 1507.2 cells within an hour (Figure 4B). Pretreatment of the cells with the ROS scavenger N-acetylcysteine (NAC) blocked curcumin induced increase of ROS levels (Figure 4B).

### NAC blocks the sensitizing effect of curcumin

Next we examined whether NAC rescues the viability of cells treated with curcumin and TRAIL. NAC completely abrogated the curcumin-induced enhancement of cell death and PARP-cleavage (Figure 4C, D). Interestingly, NAC pretreatment of 1507.2 cells did not alter TRAIL sensitivity in the absence of curcumin indicating that it specifically inhibits the effect of curcumin but does not interfere with endogenous TRAIL sensitivity (data not shown).

### ROS increase TRAIL sensitivity of MPNST cells

We also determined whether exogenous ROS (H_2_O_2_) that increases intracellular ROS levels dose dependently (Figure 4E) can mimic the effect of curcumin on TRAIL sensitivity. Indeed, pretreatment of 1507.2 cells with H_2_O_2_ potentiated the cytotoxicity of TRAIL and dose dependently increased cleavage of PARP (Figure 4F, G). Taken together this data strongly suggests that curcumin increases TRAIL sensitivity of MPNST cells via ROS production.

This is the first study to evaluate TRAIL on MPNST cells. All NF1 associated MPNST cell lines showed high expression of death receptors and were sensitivity to TRAIL. Notably the sporadic MPNST cell line was resistant to TRAIL and showed lower death receptor expression. However, this cell line may evade TRAIL mediated cell death using a mechanism which is unrelated to its NF1 status. Additional studies will be needed to clarify whether sporadic MPNST cells with wild type NF1 are in general less sensitive to TRAIL.

Reduced TRAIL sensitivity after re-expression of the NF1-GRD in neurofibromin deficient MPNST cells suggests that loss of neurofibromin supports TRAIL sensitivity rather than mediating resistance.

Reduced sensitivity correlated well with reduced death receptor expression and knockdown of death receptors lead to a significantly decreased sensitivity to TRAIL suggesting that death receptors are functionally relevant targets for neurofibromin.

However, we cannot exclude other regulatory points in the TRAIL signaling cascade affected by neurofibromin. An in depth analysis of the signaling events downstream of death receptors including extrinsic and intrinsic apoptotic pathways will be necessary to fully uncover the mechanistic influence of neurofibromin and its regulatory target pathways PI3K and MEK on TRAIL sensitivity in MPNST cells.

The data shows a strong dependence of death receptor expression and TRAIL sensitivity on c-MYC. We showed here for the first time that re-expression of the NF1-GRD downregulated c-MYC and upregulated MAD1. Inhibitor data suggests that inhibition of both PI3K and MEK contribute to the regulation of c-MYC and that PI3K inhibition is especially important for regulation of MAD1. Moreover knockdown of MAD1 partly rescued TRAIL sensitivity under PI3K inhibition strongly suggesting that upregulation of MAD1 contributes to reduced TRAIL sensitivity upon PI3K inhibition. Taken together the data suggest that neurofibromin influences TRAIL sensitivity at least in part by modulating the MYC/MAX/MAD network and that this network may be an important downstream target of deregulated RAS signaling in NF1 associated tumors. It will be interesting to determine gene promoter targets for c-MYC and MAD1 in MPNST cells and to evaluate their activity in dependence of PI3K and MEK pathways, not only to further support their contribution to TRAIL sensitivity but also to tumor cell proliferation and angiogenesis.

To use apoptosis induction via death receptors in NF1 associated MPNST to full capacity a combinatory treatment will probably be necessary. To this end we found the nontoxic phytochemical curcumin to be efficient in further increasing the sensitivity of NF1 associated MPNST cells to TRAIL. Increase in sensitivity was correlated with increased ROS levels. Moreover the sensitizing effect of curcumin was abolished by anti-oxidant treatment and mimicked by exogenous ROS strongly suggesting ROS production to be critical. There is accumulating evidence that ROS play an important role in the biology of tumor cells and that ROS could be critical for sensitizing tumor cells to TRAIL [41]–[43]. The mechanism by which ROS alters TRAIL sensitivity of MPNST cells remains to be determined. Here, it would be of special interest to closely examine the mitochondrial mediated apoptotic pathway and cellular stress pathways involving the p38 MAPK and JNK-kinases which are known to be activated by ROS [44].

Interestingly, tumors cells with hyperactive RAS signaling have been found to be especially sensitive to ROS elevating agents and in a drosophila model neurofibromin deficiency was associated with increased ROS generation [45], [46]. This may open up the option to take advantage of NF1 loss in tumor cells in two ways. One by using TRAILs tumor specificity to which RAS likely contributes in MPNST and the other one by combining it with an agent that induces ROS preferentially in tumor cells like curcumin.

There are several ongoing clinical trials (phase I/II/III) to evaluate the efficiency of curcumin in different types of cancer. In the moment the poor bioavailability of curcumin in certain tissues still limits its usefulness as therapeutic agent. However, development of means to overcome this limitation is an area of intensive research. One strategy uses nanoparticles to increase the bioavailability of curcumin [47]. Furthermore, several analogues of curcumin with enhanced biological activity (biocurcumax, diphenyldifluoroketone) have been found and are currently in preclinical and clinical evaluation [48], [49]. Alternatively other agents that induce ROS production in tumor cells may represent an option for the treatment of NF1 associated MPNST in combination with TRAIL in the future.

## Supporting Information

Figure S1 A, S462, ST88-14 and NFS-1 cells were pretreated with DMSO or the inhibitors LY294002 (20 µM) or U0126 (20 µM) for 24 h and subsequently treated with 100 ng/ml TRAIL for 20 h. Viability was analyzed by crystal violet assay. The calculated additive effect of cotreatment (TRAIL and inhibitor) was determined and compared with the actually observed effect. Corresponding immunoblots show phosphorylation levels of ERK and AKT and expression of death receptors (DR4, DR5). The values below the bands are the relative densities. B, Proteome Profiler apoptosis array. Upper membrane shows 1507.2 cells incubated with curcumin (10 µM), lower membrane shows 1507.2 cells incubated with DMSO.
(TIF)
