# Supplementary figures and images for: Sensitivity of Malignant Peripheral Nerve Sheath Tumor Cells to TRAIL Is Augmented by Loss of NF1 through Modulation of MYC/MAD and Is Potentiated by Curcumin through Induction of ROS
Source: PLoS One. 2013 Feb 21;8(2):e57152. doi: 10.1371/journal.pone.0057152 (PMC3578816; doi:10.1371/journal.pone.0057152)

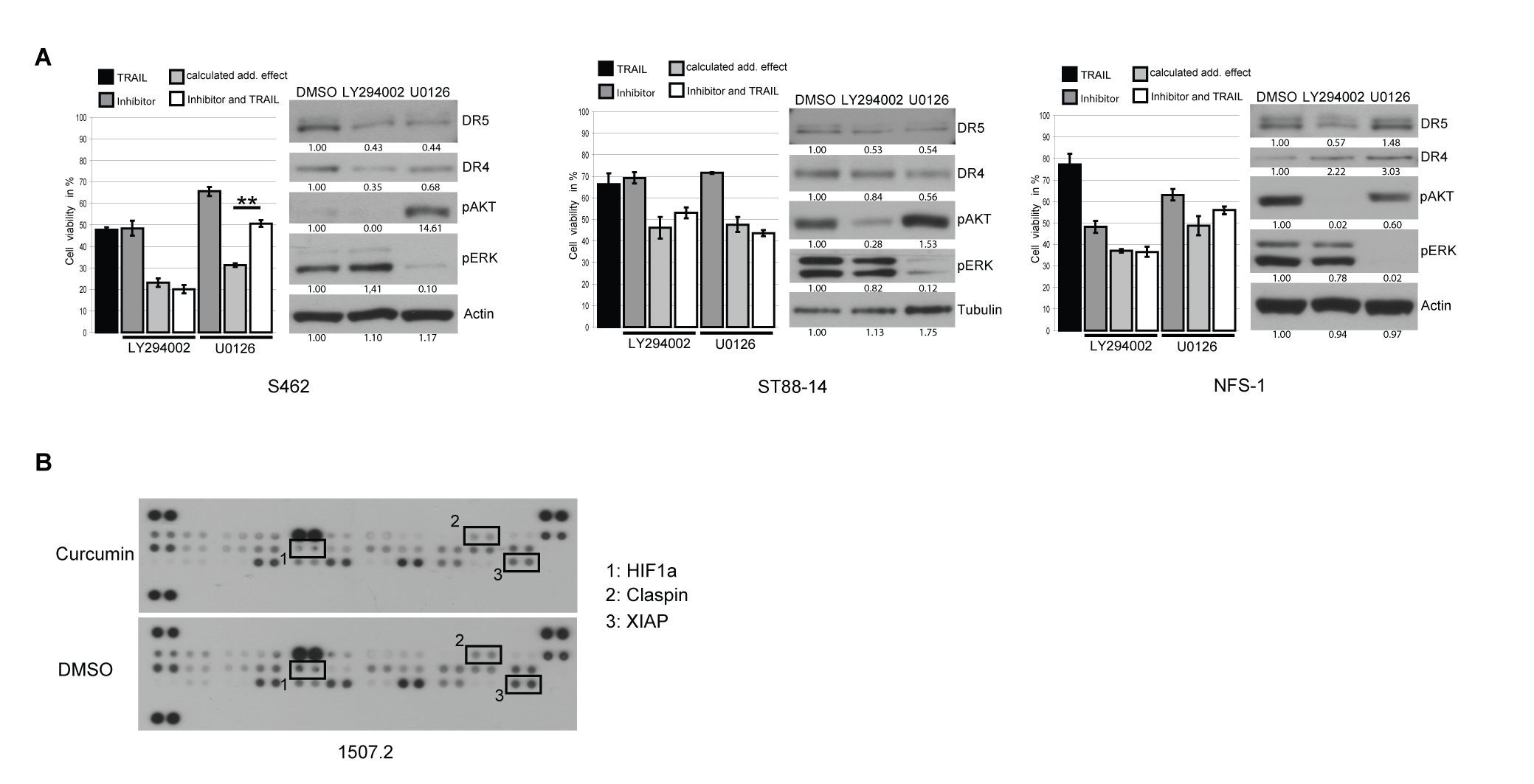

Supplement: Figure S1 — A, S462, ST88-14 and NFS-1 cells were pretreated with DMSO or the inhibitors LY294002 (20 µM) or U0126 (20 µM) for 24 h and subsequently treated with 100 ng/ml TRAIL for 20 h. Viability was analyzed by crystal violet assay. The calculated additive effect of cotreatment (TRAIL and inhibitor) was determined and compared with the actually observed effect. Corresponding immunoblots show phosphorylation levels of ERK and AKT and expression of death receptors (DR4, DR5). The values below the bands are the relative densities. B, Proteome Profiler apoptosis array. Upper membrane shows 1507.2 cells incubated with curcumin (10 µM), lower membrane shows 1507.2 cells incubated with DMSO. (TIF) [file pone.0057152.s001.tif]
